# Development and Validation of a Virtual Gelatin Model Using Molecular Modeling Computational Tools

**DOI:** 10.3390/molecules24183365

**Published:** 2019-09-16

**Authors:** Lukasz Radosinski, Karolina Labus, Piotr Zemojtel, Jakub W. Wojciechowski

**Affiliations:** 1 Department of Bioprocess and Biochemical Engineering, Faculty of Chemistry, Wroclaw University of Science and Technology, 50-370 Wroclaw, Poland; 2 Faculty of Chemistry, Wroclaw University of Science and Technology, 50-370 Wroclaw, Poland; piotr.zemojtel@gmail.com; 3 Faculty of Fundamental Problems of Technology, Wroclaw University of Science and Technology, 50-370 Wroclaw, Poland; kubwoj95@gmail.com

**Keywords:** biopolymers, gelatin, hydrogel, molecular dynamics, functional polymeric matrices

## Abstract

To successfully design and optimize the application of hydrogel matrices one has to effectively combine computational design tools with experimental methods. In this context, one of the most promising techniques is molecular modeling, which requires however accurate molecular models representing the investigated material. Although this method has been successfully used over the years for predicting the properties of polymers, its application to biopolymers, including gelatin, is limited. In this paper we provide a method for creating an atomistic representation of gelatin based on the modified FASTA codes of natural collagen. We show that the model created in this manner reproduces known experimental values of gelatin properties like density, glass-rubber transition temperature, WAXS profile and isobaric thermal expansion coefficient. We also present that molecular dynamics using the INTERFACE force field provides enough accuracy to track changes of density, fractional free volume and Hansen solubility coefficient over a narrow temperature regime (273–318 K) with 1 K accuracy. Thus we depict that using molecular dynamics one can predict properties of gelatin biopolymer as an efficient matrix for immobilization of various bioactive compounds, including enzymes.

## 1. Introduction

In recent years, a significant growth has been observed in the dynamics of research on naturally derived polymers [1,2,3]. Due to their widespread availability in nature and beneficial properties such as renewability, non-toxicity, biocompatibility and biodegradability, bio-origin materials have become favourable alternatives to conventional fossil-based polymers [4,5]. Currently, intensive research is focused on the field of biomaterials development. This phenomenon is the indicated response related to the increased pro-ecological consciousness of worldwide society as well as to the gradual decline in oil resources resulting in reduced availability of polymers based on petroleum derivatives [6,7]. Biopolymers of natural origin (e.g., cellulose, chitosan, agarose, carrageenan, alginate, pectins, gelatin, milk proteins, etc.) are huge group of multipurpose organic compounds of diverse functional properties, based on the specificity of the reactive groups present in their structure. This all contributes to the fact that renewable polymeric biomaterials are currently being successfully utilized in a variety of medical [8,9,10], pharmaceutical [11,12,13], food processing [3,14,15,16,17], sensing [18,19,20,21], agricultural [22,23], environmental protection [24,25,26] and other biotechnological applications. 

Moreover, the use of hydrogels based on natural-derived biopolymers as carriers for effective immobilization of biocatalysts is of great practical interest [3,27,28,29,30,31,32]. The application of this type of matrices is relatively simple, cheap and does not cause severe changes in the native structure of enzymes after immobilization, which enable them to retain an appropriate level of catalytic properties. Most of all, the significant merits of hydrogel-based natural carriers are their ability to create multilayer and semipermeable systems, similarity to natural tissues, green/pro-environmental character and the possibility of simple modification of their functional properties by changing the ratio of individual building blocks and/or introducing new components into their structures [33,34,35].

To date, most studies on obtaining hydrogels with the desired properties have been performed only experimentally—generally using the so-called trial and error method [33]. As a final result, this pathway can be fairly effective, but its execution is long, tedious and requires outstanding practical experience. Taking into account the time and costs of experimental research, it would be much more efficient and economical to use computational tools enabling virtual design of a three-dimensional hydrogel network with specific structural properties, i.e., crosslinking degree, pore size, and mechanical strength. In this way, it would be possible to design hydrogel materials with given characteristics required for a particular application without the necessity to perform a series of experimental preliminary tests. Moreover, it is anticipated, that thanks to this solution, it would be feasible to restrict the range of concentrations of individual reagents (such as: hydrogel building blocks, or cross-linking agents) considered during practical research on preparation of materials with the desired properties. 

The subject of our research is gelatin—the product of partial acidic or alkaline hydrolysis of collagen—which consists of peptide chains of varying length [36]. Due to its biocompatibility, biodegradability, high availability and low cost this animal-originated biopolymer is widely used in variety of practical applications. The multi-functionality of gelatin is also determined by numerous active moieties located on its polypeptide chains, which are potential crosslinking and docking sites for a variety of desirable functional molecules [37,38,39]. Furthermore, this biopolymer is considered as Generally Recognized As Safe (GRAS) by the United States Food and Drug Administration (FDA) [40]. That makes it the material of choice in many targeted applications in the food, cosmetics, pharmaceutical and medical industries [40,41,42,43]. Although there are numerous experimental studies concerning the properties and utilization of gelatin, papers analysing its features using molecular modeling are few at most [44,45,46,47]. One probable reason is the difficulty in creating a reliable atomistic model due to the complex nature of gelatin as a biopolymer. Natural occurring gelatin consists of 13 semi-randomly ordered units of amino acids and thus creating reliable yet efficient models is difficult. The most detailed studies are those performed by Zaupa et. al. [45,46] where they studied functionalized gelatin models with desaminotyrosine (DAT) and desaminotyrosyl-tyrosine (DATT). Using a combination of classical methods like Molecular Dynamics [48] and Monte Carlo [49] they related the level of functionalization with the swelling degree and mechanical properties of gelatin. In turn, Knani et al. [47] studied novel porous soy protein conjugates with gelatin and alginate. Using molecular dynamics they calculated the mixing energies between soy and gelatin and alginate. By calculating the radial distribution function they estimated the interaction distance between the compounds and crosslinking agent and thus crosslinking degree. 

In order to optimize the utilization of a biopolymer matrix one has to have a method of relating its chemical and physical characteristics with the individual properties of the immobilized compound. Since in our research studies we focused on using gelatin and its derivatives as a carrier for enzyme immobilization, the crucial factors that determine its application are related with mechanical, structural and transport features of the investigated bio-matrix. The activity of the enzyme is directly related with the diffusion of the substrates and products through the polymer net, whereas its stability is related to the mechanical and energetic properties. Molecular modeling approaches may thus offer the possibility of directly relating the chemical structure of such a bio-matrix with its applications, in this case enzyme immobilization. The fundamental problem in this approach is a creation of a proper molecular model of the gelatin that could be effectively used to reproduce the desired properties of materials based on this biopolymer.

The main roadmap of the research is to create a method that combines both experimental data and simulations to optimize the application of a hydrogel matrix as an enzyme immobilization carrier. To fulfill this goal one needs to know the operational parameters of the matrix (density, pore distribution, transport and mechanical properties, reactivity, swelling, etc.) as a function of temperature and the chemical properties of the matrix itself modified by the crosslinks and functional groups. In molecular simulations the first step is to create a reliable atomistic model. The model has to be then validated by the agreement of chosen observables calculated in simulations with experimental values. In our paper we choose such observables as density (giving the rough estimation of packing and separation of mass of polymer in the given volume), glass-rubber transition temperature, thermal expansion coefficient, Hansen solubility parameter and wide angle X-ray scattering (WAXS) profiles. We postulate that the gelatin model proposed in our research will also give the possibility of tracking structural changes like specific volume and fractional free volume in the temperature operational range of the considered enzyme. In order to provide simulation data including solvent effects explicitly one has to use coarse-grained simulations like dissipative particle dynamics (DPD). In this approach, the definition of the super-atom structure (beads) is given by the definition of atomistic model of the investigated polymer and the interactions between super-atoms (beads) are a function of the Hansen solubility parameter. Once the DPD model is created, it is possible to measure the pore distribution and other properties in the presence of solvent and compare it with experimental values. Due these facts the choice of the initial atomistic model of gelatin and validation of structural (density, WAXS), energetic (Hansen solubility parameter) and thermal (isobaric thermal expansion coefficient) parameters iare crucial steps towards study of hydrogel in the presence of solvent and eventually an immobilized enzyme in a given range of operational temperatures. 

We intend to show that molecular dynamics as applied to our model provides enough accuracy to predict some fundamental properties of gelatin as a polymer, like density, glass-rubber transition temperature, isobaric thermal expansion coefficient and WAXS profile. Then by varying temperature from 273 to 318 K we are able to track changes of its density, fractional free volume (FFV) and Hansen solubility parameter. In the following studies, it is planned to include solvent effect using our previously validated gelatin model.

## 2. Results

### 2.1. Structural Model of Gelatin

In this section we propose a novel method to create a virtual model of gelatin fibers from collagen PDB structures as well as various results verifying the correctness of our model. Gelatin is an important material in biotechnology, medicine and food industry. Thus one requires detailed studies of its mechanical, transport, sorption and chemical properties. Among a number of methods available to analyze the aforementioned features molecular modeling is one of the most versatile. In order to apply molecular modeling techniques like ab initio, Molecular Dynamics, Monte Carlo and eventually coarse-grained methods like DPD, one need first an atomistic model of the gelatin structure. Unfortunately the literature or available structural databases do not provide a detailed gelatin sequence. Thus in our work we have developed a procedure to create such a model using combination of currently available software supported by scripts written in Python programming language.

The procedure starts by importing the human-derived α-1 collagen fiber FASTA code from the Uniprot database. It should be noted that one of the key features of the gelatin structure is a presence of hydroxylated prolines. Unfortunately this molecular residue is not present in the original FASTA code. Thus to fill this gap in our code the hydroxyproline is denoted as an X element. After this operation, the collagen string is represented by 1464 amino acids (see the Materials and Methods section).

**Figure 1:**
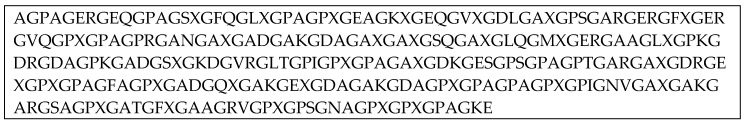
Final gelatin sequence of 280 amino acids length. Abbreviations: G—Glycine, A—Alanine, V—Valine, L—Leucine, I—Isoleucine, P—Proline, X—Hydroxyproline, S—Serine, T—Threonine, M—Methionine, F—Phenylalanine, D—Aspartic acid, N—Asparagine, E—Glutamic acid, Q—Glutamine, K—Lysine, R—Arginine.

The sequence is then divided using Python code simulating natural gelatin cleavage by trypsin. Trypsin cut peptides on the C-terminal side of lysine and arginine amino acid residues. If a proline residue is on the carboxyl side of the cleavage site, the cleavage will not occur. Once we obtain the FASTA code with an appropriate distribution of peptides the code is being translated to PDB molecular format using the Avogadro software. As a result we have obtained a gelatin chain consisting of 280 amino acids (Figure 1). Using the procedure proposed in our study, one can create gelatin fibers of any length or number of chains. 

**Figure 2:**
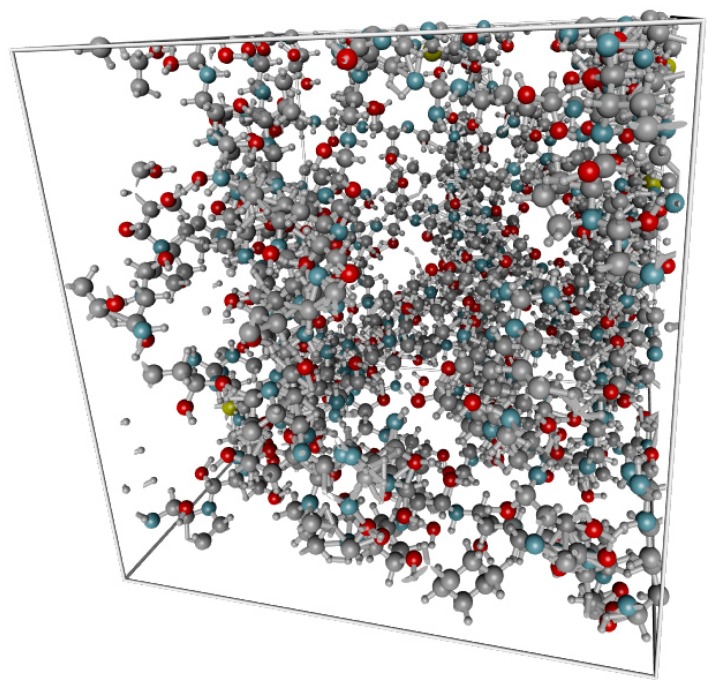
Gelatin model in cubic periodic boundary box of the size 31.89 Å (3462 atoms). The carbon atoms are colored in grey, oxygen in red, nitrogen in blue and hydrogen in white.

Having obtained the appropriate structure file, the gelatin fiber was then folded using Polymatic [50] tool and INTERFACE force field (Figure 2). 

INTERFACE is an extension of the PCFF force field using the same functional form, however, the parametrization has been majorly improved. The interesting property of the INTERFACE FF is its transferability, which give it the ability to study organic-inorganic systems. Furthermore, it was optimized to study given phenomena under various pH conditions. Thus it is the computational tool of special interest in the case of research on hydrogel matrices. Due to possibility of analyzing not only hydrogel structure but also pH dependent interfaces of complex solid state hydrogel. The detailed discussion about parametrization and properties of the INTERFACE force field may be found in our pervious study [48].

### 2.2. Veryfication of the Model

In order to verify the obtained model, the system was then equilibrated using Molecular Dynamics as implemented in the LAMMPS simulation package. The motion equations were integrated using the Velocity Verlet algorithm with a 1 fs timestep. The studied system is highly amorphous and has a large number of degrees of freedom. Therefore, it requires an equilibration protocol that prevents the system from occupying a high energy metastable state. Thus, we have derived the equilibration protocol that includes heating up and careful cooling down steps. First, the system was heated up to 600 K in the NPT canonical ensemble using Nose-Hoover thermostat and Andersen barostat (1 Pa) and then was equilibrated for 300 ps and after that gradually cooled down by 50 K in each step. In each run, the temperature and pressure were equilibrated for 50 ps until next step began. Upon reaching desired final temperature i.e., 298 K the system was equilibrated for another 500 ps in NPT ensemble and then for another 500 ps in NVT ensemble. This procedure lead to obtain the final density 1.3248 g/cm^3^ being only 2% lesser from experimental value 1.35 g/cm^3^ [51]. 

**Figure 3:**
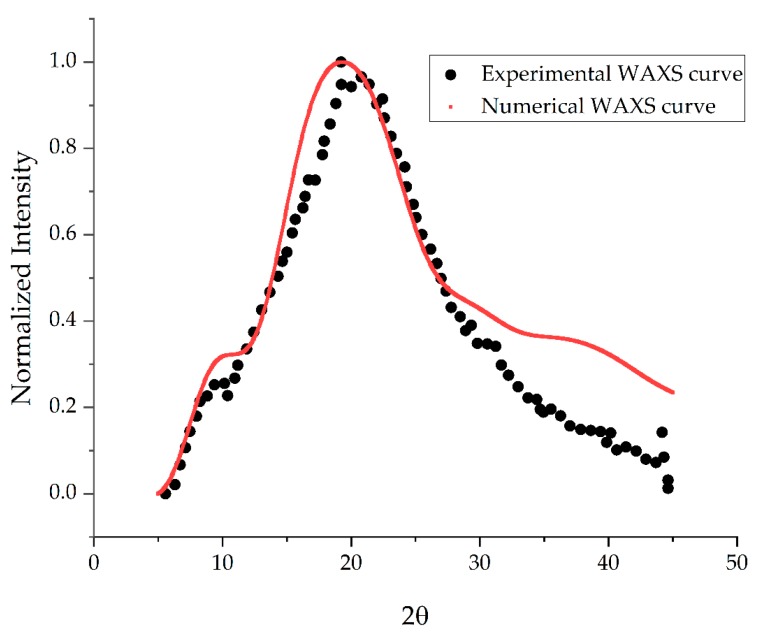
WAXS profile (λ=1.54178 Å) obtained in simulation compared with experimental one [52]. The main peak of the molecular model is at 19.39° being slightly different from natural dry gelatin (22.4°).

To further investigate the quality of our model we have calculated the WAXS profiles in the range of 5 to 50 degrees of the 2θ angle (Figure 3). The major peak of WAXS profile of the model is located at 19.39°. This value is slightly larger from that of natural dry gelatin (22.40°) suggesting that gelatin fibers are separated a little more from each other than in the natural state. That conclusion corresponds well with a little lesser density of virtual model of gelatin proposed in this study.

**Figure 4:**
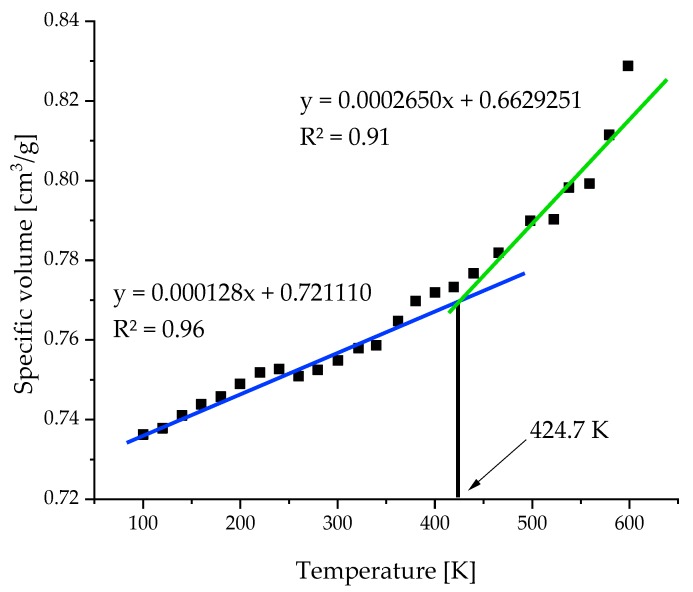
Temperature dependence of specific volume. At 424.7 K there is clear change of curvature of the dependency indicating glass-rubber transition.

By calculating the change of specific volume being defined as the inverse of the density, one may also estimate glass-rubber transition (*T_g_*). By performing series of NPT-MD simulations in the temperature range of 100 to 600 K we estimated *T_g_* as 424.7 K (Figure 4). One has to point out is that the experimental result concerning the *T_g_* of dry gelatin is not well established as an exact value. There are numerous experimental values given in the literature [53], ranging from 368 K to 473 depending on the chosen method (dilatometry, viscoelastic, DTA). The most reliable result seems to be 448 ± 10 K reported in the Polymer Data Handbook [53] which is very close to our result (424.7 K). Such agreement indicates that our model, besides properly mapping structural changes, may also reproduce thermal coefficients like heat capacity or isobaric thermal coefficient.

### 2.3. Temperature Resolution of the Model

Since in our study the crucial factor is the temperature resolution of the model we studied the change of temperature in 273–318 K regime with 1 K temperature increment. One of the characteristic values for polymeric identifying its thermal properties if the isobaric thermal coefficient defined as:

(1) $$\alpha_{p}=\frac{1}{V}{\left(\frac{\partial V}{\partial T}\right)}_{p}$$

where *V* is the volume and *T* is the temperature. To the best of our knowledge, the isobaric thermal coefficient of the solid gelatin is unknown. Taffel [54] calculated the mean isobaric thermal coefficient of the 25% gelatin mixture in water in the temperature range 273–318 K as 3.864 × 10^−4^ 1/K. As indicated in his work, assuming a linear relationship of gelatin thermal expansion coefficient with water concentration, its value obtained for dry gelatin is 8 × 10^−4^ 1/K. In our simulation we calculate the density (ρ) of the system. Nevertheless, using the fact that ρ=M/V where *M* is the total mass of the system and *V* its volume the equation can be easily transformed into:

(2) $$\alpha_{p}=-{\left(\frac{\partial ln\rho }{\partial T}\right)}_{p}$$

**Figure 5:**
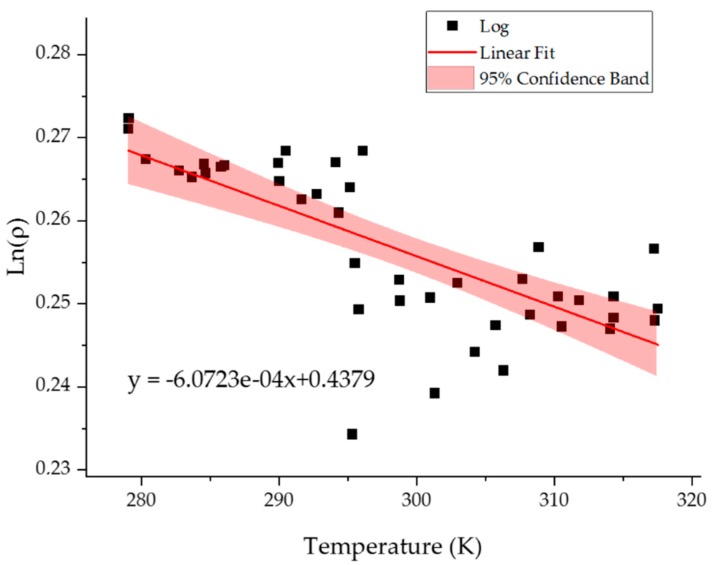
The dependence of Lnρ on temperature. The red areas denote 95% confidence bands. The value of the slope is $-6.0723*10^{-4}\pm 8.8*10^{-5}\frac{1}{K}$.

Thus by measuring the slope of the lnρ(T) function one can calculate the isobaric thermal expansion coefficient (*α_p_*) (Figure 5). to the value obtained as a result of our study (6.072 × 10^−4^ 1/K) is being very close to the value predicted by Taffel (8 × 10^−4^ 1/K) [54].

The ANOVA analysis (Table 1) indicates that although large variations in the value of the Lnρ values around the line there is enough data to postulate linear change of the Lnρ with temperature.

At the final stage of our analysis of developed gelatin model we have calculated the changes of Hansen solubility parameter and fractional free volume with temperature. The Hansen solubility parameter δ is defined as:

(3) $$\delta =\sqrt{CED},$$

where CED is the cohesive energy density of the system.

The Hansen solubility parameter was computed by dividing the value of the total non-bonded energy predicted by the INTERFACE force field *E_i_* by the actual volume *V_i_* of the unit cell in the simulation in every frame of the simulation and averaging over the number *N* of frames in the NPT equilibrium regime:

(4) $$\delta \left(T\right)=\frac{\sum_{i=1}^{N}\sqrt{\frac{E_{i}^{nb}\left(T_{i}\right)}{V_{i}\left(T_{i}\right)}}}{N}$$

**Figure 6:**
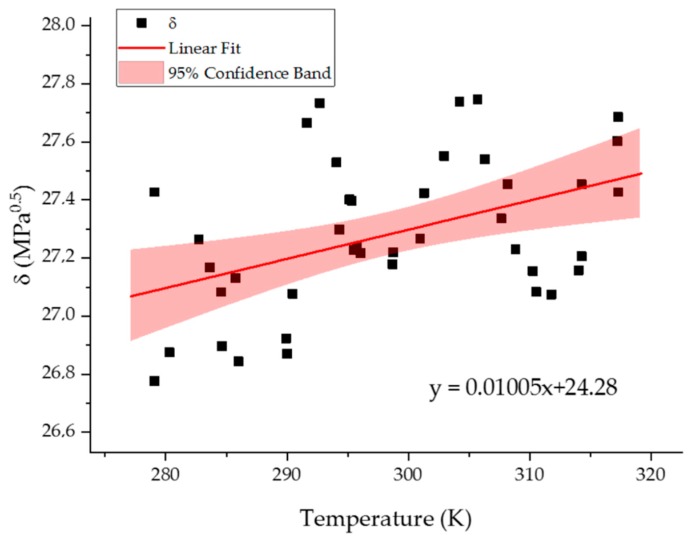
The dependence of Hansen solubility parameter on temperature. The value of the slope of linear correlation (red line) is $0.01005\pm 0.0032\frac{MPa^{0.5}}{K}$. The red areas denote 95% confidence intervals.

The results are presented in Figure 6.

Our model indicates that the Hansen solubility parameter for 298 K 27.2 ± 0.05 MPa^0.5^. This value is a little higher than the one obtained by Zaupa et al. [46] (25.02 ± 3.6 MPa^0.5^). However, his model included 0.8% of water resulting in smaller density of the system namely 1.233 g/cm^3^. Due to larger separation between chains in the Zaupa model, such differences may be expected. We also calculated the change of δ with temperature (Figure 6). The ANOVA test (Table 2) also in this case indicated that tracking the change of δ using our model is possible.

**Figure 7:**
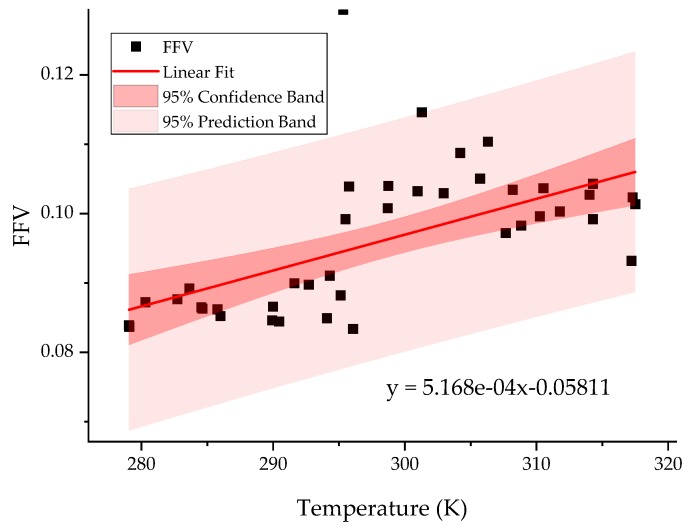
The dependence of FFV on temperature. The value of the slope of linear correlation (red line) is $5.168*10^{-4}\pm 1.09*10^{-4}\frac{1}{K}$. The red and pink areas denote 95% confidence and prediction intervals respectively.

The fractional free volume (FFV) is defined as:

(5) $$\;FFV=\frac{V_{total}-V_{gelatin}}{V_{total}}$$

where $V_{total}$ is the total volume of the simulation box and $V_{gelatin}\;$ is the volume of space occupied by the polymer. Thus by calculating the volume of the simulation box and gelatin in every frame in equilibrium one can easily calculate the dependence of FFV on temperature (Figure 7) as:

(6) $$FFV\left(T\right)=\frac{\sum_{i=1}^{N}FFV_{i}\left(T_{i}\right)}{N}$$

Moreover, as one can see from ANOVA analysis (Table 3) our model provides enough statistical evidence to track changes of FFV in the operational temperature range of the enzyme in such cross-linked gelatin matrix. 

## 3. Discussion

The typical approach for studying and optimizing the applications of gelatin is a biochemical one, where gelatin is treated as a protein derivative and its properties are mainly governed by the different kinds of functionalization. In our complementary approach, we consider gelatin from the point of view of polymer science. In this context, the properties of the material can be described by several factors that can be directly measured in experiments. These parameters are related with desired functional properties—(e.g., permeability, swelling, thermal expansion, miscibility) being defined by self-diffusion coefficient, fractional free volume, Hansen solubility and others. Such calculations, using molecular modeling, are widely used not only in science but also in industry because they connect the values of the aforementioned parameters with the physicochemical environment of the polymer and thus offer the possibility to tune its performance. Although gelatin is by definition a polymer this approach is rarely used to study and optimize its properties. 

Due to the fact that the overriding aim of our research is to study how the physicochemical properties of a biopolymer matrix affects the catalytic features of a given enzyme (its activity and stability) in a given operational temperature range (namely 273–318 K), in this case, we intend to use this polymeric approach to develop virtual model of gelatin that could be applicable for further designing the functional hydrogel materials characterized by strictly defined structural properties. 

One of the limiting factors of enzyme activity is the intake of substrate in the vicinity of the active site and the expulsion of product. This phenomenon is directly related by the diffusion of these both reactants. One of the fundamentals factors affecting diffusivity in porous materials was previously defined by fractional free volume (FFV) (Equation (5)). The mobility of substrate/product can be characterized by the self-diffusion coefficient related with FFV by the Fujita model:

(7) $$D=D_{0}RTAe^{-\frac{B}{FFV}}$$

where *D* is the self-diffusion coefficient of the small molecules penetrating in the polymeric solid, *D*_0_ is the pre-exponential diffusion value when the penetrant concentration is 0. This equation can be easy linearized by applying log function resulting in:

(8) $$\log D=C+T-\frac{B}{FFV}$$

where $C=\log D_{0}+\log R+\log A$. In order to compute the model one has to calculate self-diffusion coefficient vs. FFV at different temperatures. 

Another important issue is the Hansen solubility parameter δ [55]. The importance of this coefficient rises from Flory-Huggins and Flory-Rehner [46] theory that describes the miscibility of given polymer with other polymers or solvents as well as the equilibrium swelling ratio by means of the Hansen solubility parameter. By computing the value of δ for polymer (gelatin) and solvent (water) one can calculate so callaed χ interaction parameter using formula:

(9) $$\chi_{sp}=\frac{V}{RT}{\left(\delta_{s}-\delta_{p}\right)}^{2}$$

Then using the Flory-Rehner equation one can calculate how the equilibrium sweling degree is changing due to crosslinking level, functionalizatino or change of operational parameters of the process. The Flory Rehner equation is given by:

(10) $$d=\frac{\left[Ln\left(1-v_{s}\right)+v_{s}+\chi v_{s}^{2}\right]}{V_{sol}\left[v_{s}^{\frac{1}{3}}-\frac{2v_{s}}{\phi }\right]}$$

where $v_{s}$ is the polymer volume, ϕ is the polymer molar fraction, *d* is the crosslinking density, $V_{sol}$ is the molar volume of the solvent. Thus the equilibrium swelling can be computed as:

(11) $$Q=\frac{1}{v_{s}},$$

The method presented in the paper offers possibility to directly compute aferomentioned parameters. If however one wants to study solvent effects explicitly in order to track the FFV distribution or study transport phenomena in the presence of an immobilized enzyme the swelling nature of the hydrogel requires large systems exceeding 100,000 atoms or more. In such case DPD or other coarse-grained methods seem like a natural choice. However as indicated before an initial atomistic model of the system is also required together with interaction parameters being a function of Hansen solubility parameter.

Moreover statistical analysis shows that data provided by the molecular simulations are sufficient to study the thermal evolution in such narrow regime (273–318 K). That information is essential from the point of view of further studies of activity and stability of the bioactive element implemented in cross-linked biopolymer structure. As presented in Section 2, the F-tests indicated that we managed to observe significant changes of parameters over a very narrow temperature range and thus create a reliable linear model. 

Summing up the performed research, in this paper we have presented a method for creating an effective atomistic model of gelatin using collagen models and computer simulation of its partial enzymatic decomposition to gelatin fibers catalyzed by trypsin. The proposed method combines simple operations on FASTA strings with Monte Carlo and Molecular Dynamics Simulations. Subsequently we have shown that our atomistic model of gelatin combined with the INTERFACE force field and the NPT Molecular Dynamics method is capable to predict various energetic and structural properties characteristic for polymers like density, glass-rubber transition temperature and isobaric compressibility coefficient. Furthermore we indicate that our method is valid for tracking structural changes over a narrow temperature range and energetic changes by means of fractional free volume and Hansen solubility coefficient, and thus providing additional insight into further optimization of application of gelatin-based carrier for enzyme immobilization. 

## 4. Materials and Methods 

**Figure 8:**
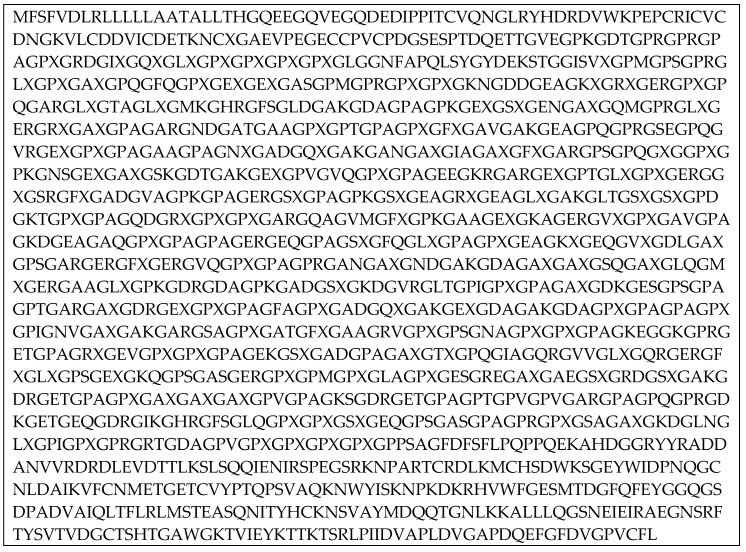
FASTA code of a collagen chain composed of 1464 amino acids. Abbreviations: G—Glycine, A—Alanine, V—Valine, L—Leucine, I—Isoleucine, P—Proline, X—Hydroxyproline, S—Serine, T—Threonine, C—Cysteine, M—Methionine, F—Phenylalanine, Y—Tyrosine, W—Tryptophan, D—Aspartic acid, N—Asparagine, E—Glutamic acid, Q—Glutamine, K—Lysine, R—Arginine, H—Histidine.

The FASTA code of collagen alpha-1 helix has been imported from Uniprot database. The string was then transformed into the final one letter code of the amino acid sequence using Python 2.7 scripts (Figure 8). 

**Figure 9:**
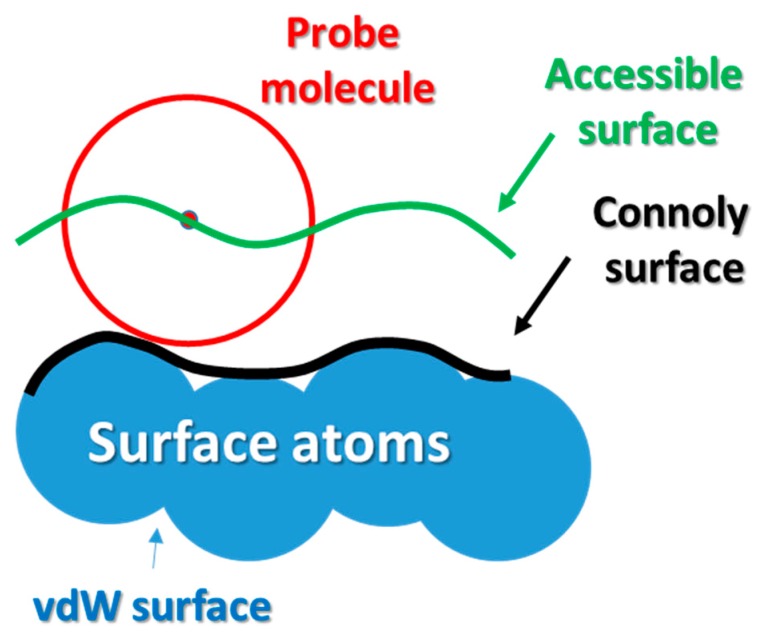
Graphical representation of the Connolly and accessible surface. The probe molecule represented as sphere of given radius is being rotated on the surface defined by the van der Walls spheres of the constituent elements of given material. The center of probe molecule defines the Connolly surface.

The FASTA code was then translated into a pdb file using the Avogadro 2.0 software under a free user license. The pdb file of the gelatin model was transformed into a 3D amorphous matrix with periodic boundary conditions using the Polymatic software under the nanoHUB web application. The cubic simulation box has 31.89 Å in every dimension and contains 3462 atoms. All the NPT Molecular Dynamics simulations were carried out in LAMMPS molecular dynamics package using the INTERFACE force field. The value of the free volume was computed using the iRASPA package using an algorithm firstly introduced by Connolly [56]. As presented in Figure 9 the Connolly and accessible surface, the probe molecule (represented as red sphere of given radius) was rotated on the surface defined by the van der Walls spheres of the constituent elements of given material. The center of the probe molecule defines the accessible surface.

**Figure 10:**
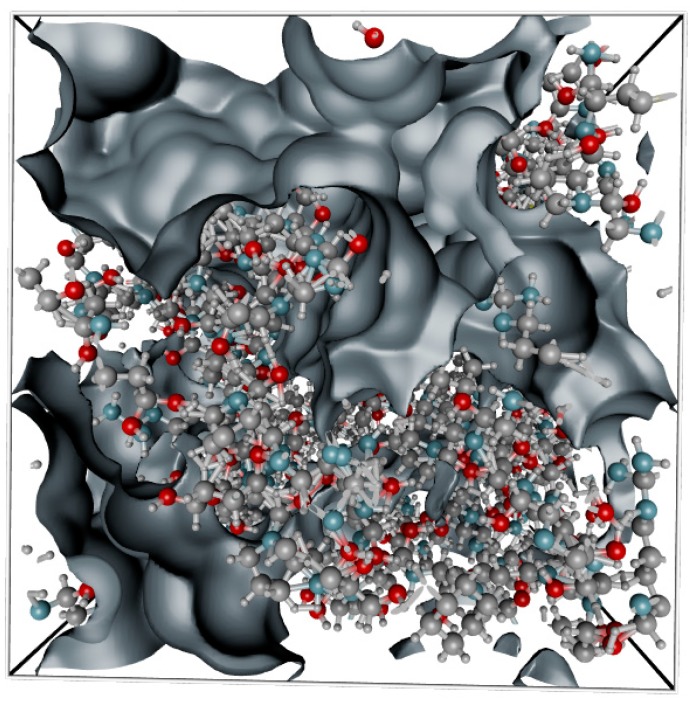
The grey surface indicate free volume surface created by the water molecule probe. The carbon atoms are colored in grey, oxygen in red, nitrogen in blue and hydrogen in white.

Figure 10 presents the Connolly and accessible surface calculated using water as probe molecule. As one can observe the occupied volume in the system is much greater than a simple sum of the vdW spheres of the constituent atoms. By subtracting the occupied volume from the simulation box volume one can calculate the free volume and eventually compute the fractional free volume (FFV).

The WAXS intensity was calculated by projecting interatomic position vector ${\bar{r}}_{ij}$ on the scattering vector *Q* using following equation:

(12) $$I\left(Q\right)=\;\sum_{kj}f_{k}\left(Q\right)f_{j}\left(Q\right)e^{i{\bar{r}}_{kj}Q},$$

where:

(13) $$Q=\frac{2\sin\theta }{\lambda },$$

The θ is the scattering angle and λ=1.54178 Å is the wave length of X-ray radiation. 

## Figures and Tables

**Table 1:** ANOVA table for Lnρ(T) model indicating that non-zero slope value hypothesis cannot be rejected (DF—degrees of freedom, SSE—Sum of Squared Errors, MSE—Mean Squared Error).

		DF	SSE	MSE	F Value	*p*-Value
Lnρ	Model	1	0.00211	0.00211	47.56219	2.61381 × 10^−8^
	Error	40	0.00177	4.42581 × 10^−5^		
	Total	41	0.00388			

**Table 2:** ANOVA table for δ(T) model indicating that non-zero slope value hypothesis cannot be rejected, (DF—degrees of freedom, SSE—Sum of Squared Errors, MSE—Mean Squared Error).

		DF	SSE	MSE	F Value	*p*-Value
δ(T)	Model	1	0.54061	0.54061	9.68304	0.00347
	Error	39	2.17741	0.05583		
	Total	40	2.71802			

**Table 3:** The ANOVA table for *FFV*(*T*) model indicating that non-zero slope value hypothesis cannot be rejected, (DF—degrees of freedom, SSE—Sum of Squared Errors, MSE—Mean Squared Error).

		DF	SSE	MSE	F Value	*p*-Value
**FFV**	Model	1	0.00153	0.00153	22.45198	2.71 × 10^−5^
	Error	40	0.00272	6.79 × 10^−5^		
	Total	41	0.00424			
